# Heterotopic Pancreas within the Proximal Hepatic Duct, Containing Intraductal Papillary Mucinous Neoplasm

**DOI:** 10.1155/2015/816960

**Published:** 2015-02-22

**Authors:** Alistair J. Lawrence, Aducio Thiessen, Amy Morse, A. M. James Shapiro

**Affiliations:** ^1^Department of Surgery, University of Alberta, Edmonton, AB, Canada T6G 2B7; ^2^Department of Pathology, University of Alberta, Edmonton, AB, Canada T6G 2B7; ^3^Department of Medicine, University of Alberta, Edmonton, AB, Canada T6G 2B7

## Abstract

We report a unique first case of benign heterotopic pancreas arising within the proximal hepatic bile duct, containing a focus of intraductal papillary mucinous neoplasm (IPMN). The condition was diagnosed on pathological explant after left hepatic lobectomy with total extrahepatic bile duct excision.

## 1. Introduction

The condition of heterotopic pancreas is a relatively common finding, where small foci of pancreatic rest tissue arise in intra-abdominal sites other than the pancreas. First described in 1727, aberrant pancreatic tissue is defined as ectopic location of pancreatic tissue without vascular, neural, or anatomic connections to the anatomically normal pancreas [1]. Found anywhere along the gastrointestinal tract heterotopic pancreatic tissue is typically situated in the stomach, duodenum, and jejunum [2]. Rarely, heterotopic pancreas may arise in the biliary tree or in the liver. In an autopsy study of 1000 livers, 4.1% contained intrahepatic heterotopic pancreas [3]. The majority of these were contained within the portal tracts not the hepatic ducts. Furthermore, the likelihood of developing IPMN within heterotopic pancreatic tissue is a rarity. Review of the literature revealed only 8 previous case reports of IPMN in heterotopic pancreatic tissue; none of these IPMN cases arose in heterotopic pancreatic tissue within the liver or hepatic ducts [4–11]. Therefore, to the best of our knowledge, this case represents the first attempt to report heterotopic pancreatic tissue and focus of IPMN within the biliary tree.

## 2. Case Report

**Figure 1:**
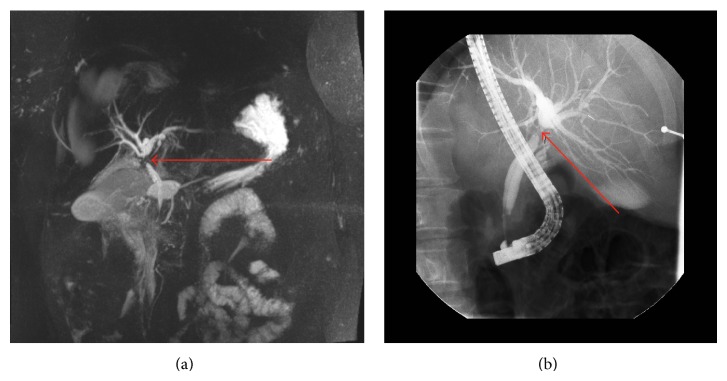
(a) Magnetic resonance cholangiopancreatography (MRCP) and (b) endoscopic retrograde cholangiopancreatography (ERCP) demonstrating stricture and occlusion of the proximal main hepatic duct (marked with red arrow).

**Figure 2:**
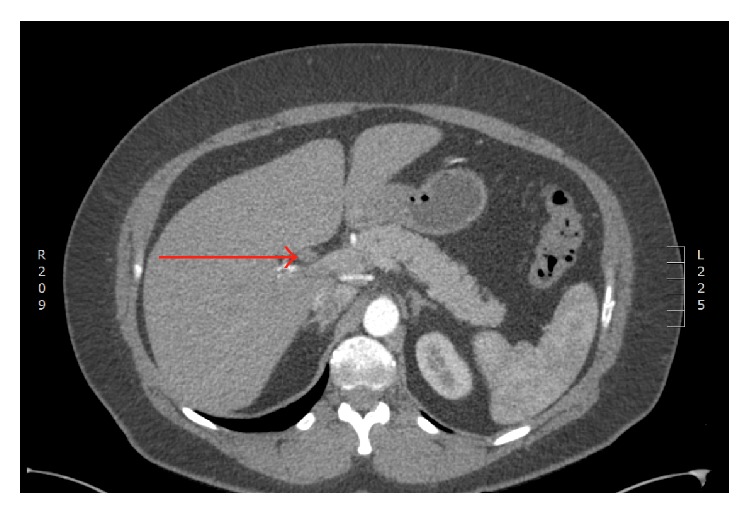
Contrast-enhanced computed tomography (CT) scan demonstrating an arterially enhancing filling defect within the proximal hepatic duct (marked with red arrow).

An obese otherwise healthy 47-year-old female (body mass index 36) presented with intermittent nausea and right upper quadrant discomfort after intentional weight loss of 27 kg. The serum lipase was found to be mildly elevated at 132 units. Abdominal ultrasound demonstrated focal dilatation of the intrahepatic left hepatic ducts, and a subsequent contrast-enhanced magnetic resonance cholangiopancreatogram (MRCP) and endoscopic retrograde cholangiopancreatogram (ERCP) demonstrated an occlusive filling defect within the proximal main biliary tree (Figure 1). An intravenous contrast-enhanced computed tomogram (CT) demonstrated a vascular, arterially enhancing filling defect within the proximal hepatic duct, with associated proximal biliary tree dilatation (Figure 2). A SpyGlass cholangiogram (Boston Scientific, Marlborough MA, USA) then revealed a frond-like polyp within the common hepatic duct, with no evidence of stone disease. Both SpyGlass-directed brushings and a blind biopsy demonstrated benign epithelial cells but were considered nondiagnostic.

The imaging findings were concerning proximal hepatic duct cholangiocarcinoma (Klatskin tumor), although the CA19-9 was not elevated. The patient then underwent surgical exploration where a palpable filling defect was identified within the proximal hepatic duct. Intraoperative ultrasound demonstrated no additional lesions. For oncological clearance, a left hepatectomy with caudate lobectomy was completed, which required a triple right-sided bile duct Roux-en-Y reconstruction (two posterior and one anterior right bile ducts) over temporary externalized 5 French stents. No blood products were required, and no inflow occlusion was used. She had a smooth and relatively uncomplicated recovery other than a superficial wound infection.

**Figure 3:**
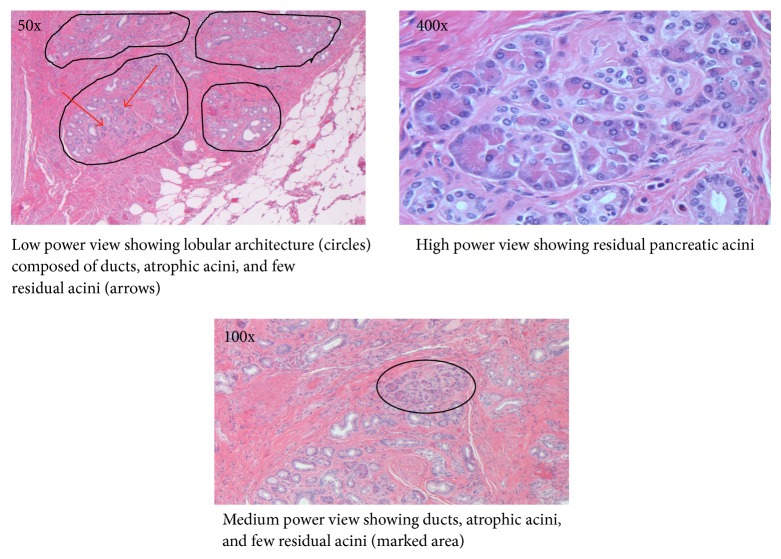
Histology of the resected left hepatic bile duct.

Surgical pathology demonstrated histological features consistent with heterotopic pancreatic tissue with IPMN contained within the proximal biliary tree (Figure 3).

Sections showed superficial atypia with areas suggestive of papillary formation. There was also prominent bland duct proliferation with lobular architecture and microscopic foci of pancreatic acini surrounding the bile duct. The histological diagnosis was consistent with heterotopic pancreatic tissue. The superficial atypia with papillae suggests a low grade IPMN of the bile duct.

At follow-up at 2 months, the patient had recovered fully, and the biliary stents were removed uneventfully.

## 3. Discussion

Continued advancement in cross-sectional and other imaging modalities facilitates early diagnosis and more precise treatment for hepatobiliary malignancies. We describe an extremely rare condition of IPMN arising within heterotopic pancreatic tissue obstructing the proximal hepatic duct. We believe this to be the first report of this condition arising within and obstructing the biliary tree. Interestingly SpyGlass cholangiography was able to demonstrate the lesion but was not able to precisely define the pathology. Furthermore, obstruction of the proximal biliary tree necessitated definitive resectional treatment. Had we been confident preoperatively of the benign nature of the filling defect, a less aggressive localized bile duct resection with hepatic parenchymal preservation could potentially have been entertained. However, the presence of IPMN change with potential for malignant transformation would still have provided concern. Preoperative diagnosis of heterotopic pancreas may be challenging and typically this is an incidental finding.

Heterotopic pancreas is most commonly found in the proximal jejunal wall of the gastrointestinal tract. Pathological changes that occur within the native pancreas may also occur rarely within the heterotopic gland, including pancreatitis, abscess, and occasionally malignant transformation [12]. IPMNs account for approximately 4% of cystic neoplasms of the pancreas [1, 13]. IPMN is a relatively new diagnosis, first reported in 1982 [14]. Clinicians should be cognisant of the fact that IPMN may arise from heterotopic pancreatic tissue and can indeed carry risk of malignant transformation. Negative margin surgical resection should therefore be entertained where appropriate for effective treatment control. Although IPMN has been reported in heterotopic pancreatic tissue, this is the first case of IPMN within the liver or hepatic ducts.

In conclusion, although the condition of heterotopic pancreas is usually asymptomatic and is often discovered incidentally, IPMN with potential for malignant transformation may rarely occur remotely within the biliary tree.
